# Regular physical activity and cardiovascular biomarkers in prevention of atherosclerosis in men: a 25-year prospective cohort study

**DOI:** 10.1186/s12872-016-0239-x

**Published:** 2016-04-05

**Authors:** Magdalena Kwaśniewska, Tomasz Kostka, Anna Jegier, Elżbieta Dziankowska-Zaborszczyk, Joanna Leszczyńska, Ewa Rębowska, Milena Orczykowska, Wojciech Drygas

**Affiliations:** Department of Preventive Medicine, Medical University of Lodz, Zeligowskiego 7/9, 90-752 Lodz, Poland; Department of Geriatrics, Medical University of Lodz, Lodz, Poland; Department of Sports Medicine, Medical University of Lodz, 92-213 Lodz, Poland; Department of Epidemiology and Biostatistics, Medical University of Lodz, Zeligowskiego 7/9, 90-752 Lodz, Poland; Central Clinical Hospital, Medical University of Lodz, 92-213 Lodz, Poland; Department of Epidemiology, Cardiovascular Disease Prevention and Health Promotion, Institute of Cardiology, Warsaw, Poland

## Abstract

**Background:**

The purpose of the study was to examine the association between leisure-time physical activity (LTPA), cardiovascular biomarkers and atherosclerosis among asymptomatic men with stable LTPA level throughout the 25-year prospective observation.

**Methods:**

Out of 101 asymptomatic men prospectively observed for their lifestyle and cardiovascular risk factors, the cohort of 62 individuals (mean age 59.9 years) maintained a stable LTPA level during the 25-year observation. Regular check-ups with the assessment of traditional risk factors, detailed measurements of LTPA level and aerobic capacity were performed since baseline. At the latest follow-up (2011/12) a set of cardiovascular biomarkers was measured using enzyme-linked immunosorbent assay. Subclinical atherosclerosis was assessed by means of coronary artery calcification score and intima-media thickness (IMT). Endothelial function was evaluated by means of the reactive hyperemia index. The studied biomarkers and indices were analyzed in the three cohorts representing stable low-tomoderate (<2050 kcal/week), high (2050-3840 kcal/week) and very high LTPA (>3840 kcal/week).

**Results:**

At baseline the three cohorts were comparable in terms of age and clinical characteristics. At follow-up, the cohort with stable high LTPA (2050-3840 kcal/week) had significantly lower concentrations of hs-CRP (2.20 ± 1.0 mg/L), oxidized-LDL (68.35 ± 67.7 ng/mL), leptin (4.71 ± 3.07 ng/mL) and irisin (0.47 ± 0.13 μmol/L), and the most favorable indices of atherosclerosis and endothelial function as compared with other groups (*p* < 0.05). Regular marathon runners had increased concentrations of hsCRP (3.12 ± 1.4 mg/L), oxidized-LDL (249.8 ± 129 ng/ml), Interleukine-6 (3.74 ± 2.4 pg/ml). A positive correlation was observed between hsCRP and IMT (*r* = 0.301; *p* < 0.01), and irisin and IMT (*r* = 0.223; *p* < 0.05).

**Conclusions:**

The data suggest that stable high LTPA (2050-3840 kcal/week) is associated with the most favorable profile of key cardiovascular biomarkers and indices of atherosclerosis. Lifetime very high LTPA is associated with increased lowgrade inflammation and may, therefore, exert an atherogenic effect.

## Background

Regular physical activity has been shown as an independent factor in prevention of cardiovascular diseases in various populations [1–5]. Current recommendations for adults indicate that meeting the minimum dose of physical activity provides substantial health benefits. It is also suggested that higher physical activity level may exert additional favorable effects [6]. There is no doubt that promoting active lifestyle remains one of the public health priorities. However, due to the growing popularity of vigorous trainings and prolonged sport events, there is also a need to investigate the health impact of exhaustive exercies.

In the Healthy Men Clinic of the Medical University of Lodz (Poland) we have a unique opportunity to follow-up the population of physically active men for over 25 years. Our previous report revealed a non-linear association between physical activity level and indices of subclinical atherosclerosis [7]. The most favorable results were found in the cohort representing intermediate physical activity level associated with energy expenditure between 2050 and 3840 kcal/week. No additional benefits were found in men regularly exceeding this dose of physical activity, and even a trend of worsening of some indices of atherosclerosis was observed. Precise mechanisms of this potentially deleterious effect of regular very high physical activity has not been documented.

It is well established that numerous potent cells and factors are involved in the process of atherosclerosis. These include C-reactive protein, cytokines, chemokines, adhesion molecules, angiogenic and other factors modifying oxidative stress and low-grade systemic inflammation [8–11]. The influence of physical activity on cardiovascular system may be associated with exercise-related modification of the above biomarkers, although precise mechanisms of this relationship remain unclear [11, 12]. Most studies in this filed adopted cross-sectional designs or short-term observation periods for the assessment of physical activity.

Therefore, the purpose of the present analysis, was to examine the association between regular physical activity level and biomarkers of cardiovascular risk in the cohort of asymptomatic men with stable leisure-time physical activity (LTPA) throughout the 25-year observation. The measurements were performed in the context of recognized indices of subclinical atherosclerosis, i.e. coronary artery calcium (CAC), intima-media thickness (IMT) and microvascular endothelial function.

## Methods

### Ethics, consent and permissions

All the subjects were provided with written information about the purpose and methodology of the study. The protocol of the project was approved by the Medical University of Lodz Ethics Committee.

### Subjects

Recruitment procedure and other methods were described in our previous papers [7, 13]. Briefly, the subjects of the study consisted of male volunteers who attended the Healthy Men Clinic and the Department of Preventive Medicine, Medical University of Lodz (Poland) since 1985. The latest follow-up was performed in 2011/12 and enrolled 101 men (mean age 59. 7 ± 9.0 yrs; mean observation period: 24.7 ± 4.1 yrs). The subjects were considered to be eligible if before the examination they were asymptomatic, free from chronic diseases and treatment (including aspirin, statins and anti-hypertensive agents) and any important disability or dementia. The whole group was initially divided into three cohorts according to the tertiles of exercise-related energy expenditure at baseline, i.e. low-to-moderate (<2050 kcal/week), high (2050-3840 kcal/week) and very high leisure-time physical activity level (>3840 kcal/week).

For the purpose of this analysis, we excluded the subjects who substantially changed their leisure-time physical activity (LTPA) level since the baseline examination (*n* = 39). Therefore, the final cohort consisted of 62 asymptomatic middle-aged and older men (mean age 59.9 ± 8.6 yrs) with stable LTPA level during the 25-year observation.

The subjects were white men, predominantly married, white collar workers with university or secondary educational level whose occupational activity was low. Majority of the study participants were non-smokers. While all the participants met current recommendations of physical activity for adults [6], a vast majority of men exceeded the minimal recommended dose of physical activity. They were involved in regular aerobic exercises (jogging, walking, cycling, tennis), including participation in marathon races.

In the years 1985-2005 all the subjects participated in a similar panel of procedures including a detailed interviewer-administered questionnaire, anthropometric and biochemical measurements, resting electrocardiogram and the graded submaximal exercise test. The latest follow-up (2011/12) included also an assessment of atherosclerosis indices and novel biomarkers.

### Assessment of traditional risk factors and physical activity

All the participants completed a medical history questionnaire which included information on their health status, family history of cardiovascular diseases, smoking, alcohol consumption and detailed information about occupational and leisure-time physical activity. The level of LTPA during the previous year was estimated. Exercise-related energy expenditure was assessed on the basis of the number of hours earmarked for weekly recreational sport activities (kcal/week) according to the tables of Fox [14].

At baseline the participants were divided into three groups according to tertiles of energy expenditure: <2050 kcal/week, 2050-3840 kcal/week, and >3840 kcal/week (low-to moderate, high and very high PA level, respectively). Next, we analyzed LTPA level throughout the whole observation. On the basis of the mean energy expenditure estimated at two-thirds of all follow-up examinations we defined subgroups of stable (maintained), increased and decreased LTPA level. For the purpose of this analysis we excluded individuals who substantially changed their LTPA level throughout the observation.

In order to assess aerobic fitness the graded submaximal exercise test was carried out on a Monark type 818E (Stockholm, Sweden) bicycle ergometer with 30 W increments every 3 min to achieve at least 85 % of maximal age-predicted HR (220-age). The resultant linear regression equation was used to calculate the aerobic capacity index, i.e. physical working capacity at 85 % of the maximal heart rate (PWC_85%HRmax_). This methodology has been proposed as a useful measure of aerobic power for epidemiological studies [15].

Fasting blood samples were drawn from the antecubital vein. Enzymatic methods were used to determine serum total cholesterol, glucose, triglycerides, uric acid concentrations (COBAS INTEGRA 400 Plus, Roche). High-density lipoproteins were measured by the precipitation method. Concentration of low-density lipoproteins was estimated using the Friedewald formula. Anthropometric data were collected by standard methods.

### Assessment of novel biomarkers and indices of atherosclerosis

#### Biomarkers

Plasma concentration of high-sensitivity C-reactive protein (hsCRP), homocysteine, interleukine-6, oxidized low-density lipoproteins (oxidized-LDL), soluble intracellular adhesion molecule-1 (sICAM-1), soluble vascular cell adhesion molecule-1 (sVCAM-1), leptin, resistin, adiponectin and irisin were measured using enzyme-linked immunosorbent assay kit (Diaclone, France; Labor Diagnostika Nord, Germany; BioVendor, Czech Republic; Immundiagnostik AG, Germany; Axis-Shield Diagnostics Ltd., Scotland). Fasting serum was stored according to the manufacturer’s recommendations. Coronary artery calcification. CAC scores were evaluated using the 64-slice computed tomography scanner (SOMATOM Sensation 64, Siemens Medical Solutions, Germany) and with Syngo CaScore automatic analysis software (Siemens Healthcare, Germany). The total CAC score was reported in Agatston units [16].

#### Intima-media thickness

Carotid IMT was measured using ultrasound scanning using the Siemens Acuson (Mountainview, CA) S2000 ultrasound system. Carotid atherosclerosis was defined as increased carotid IMT (>0.9 mm) [17].

#### Reactive hyperemia

Peripheral arterial tonometry signals were obtained using the EndoPAT 2000 device (Itamar MedicalInc., Israel). Endothelial function was assessed via RH-PAT index. RHI values <1.67 were considered abnormal [18].

### Statistical analysis

Continuous variables are expressed as mean ± standard deviation (SD) or median (if not standard distribution). Wilcoxon signed-rank test was used to assess the differences between baseline and final characteristics of the study cohorts. Categorical variables were compared by chi-square test or chi-square test with Yate’s correction. The Kruskall-Wallis test and Dunnett’s test were used in order to assess distribution of biomarkers according to the level of LTPA and to compare differences between groups. Spearman’s correlation was used to evaluate the association between biomarkers and other continuous variables. Logarithmic transformation of skewed data did not change the direction or strength of the analyzed calculations. A *p* value < 0.05 was considered statistically significant. All analyses were performed with STATISTICA Windows XP version 9.1.

## Results

Table 1 presents baseline characteristics of the whole studied group in the project (*n* = 101). Long-term changes in traditional cardiovascular risk factors of the cohorts with stable low-to-moderate, high, and very high LTPA (*n* = 62) are showed in Table 2. At baseline all the three cohorts were comparable in terms of age and clinical characteristics. At follow-up, most of the analyzed traditional risk factors changed substantially in all the groups. The LTPA level decreased during the observation, with the most striking fall in the mean energy expenditure was noticed among the participants with the highest LTPA (*p* < 0.01).

**Table 1:** Baseline characteristics of all participants of the prospective observation (*n* = 101)

	Baseline
Age, years	35.3 ± 6.4
Waist circumference, cm	85.1 ± 6.9
Body mass index, kg/m^2^	24.3 ± 2.8
Body fat, %	16.1 ± 4.2
Systolic blood pressure, mmHg	120.3 ± 12.9
Diastolic blood pressure, mmHg	78.1 ± 6.6
Triglycerides, mmol/l	1.27 ± 0.44
High-density lipoproteins, mmol/l	1.30 ± 0.28
Fasting plasma glucose, mmol/l	4.52 ± 0.56
Current smokers, *n*	17
Alcohol consumption, units/week	4.71 ± 3.6
Leisure-time physical activity, hours/week	
Low/moderate intensity (≤6 METs)	2.9 ± 2.98
High/very high intensity (>6 METs)	4.8 ± 1.9
Exercise-related energy expenditure, kcal/week	3101.3 ± 2871.8
Physical working capacity, W/kg	2.41 ± 0.56

Data presented as mean ± SD unless otherwise stated

**Table 2:** Changes in traditional cardiovascular risk factors among middle-aged men with stable physical activity level during a 25-year observation (*n* = 62)

	Stable physical activity level during the 25-year observation					
	Low/moderate (<2050 kcal/week)		High (2050-3840 kcal/week)		Very high (>3840 kcal/week)	
	*n*=26		*n*=21		*n*=15	
	Baseline	Follow-up	Baseline	Follow-up	Baseline	Follow-up
Age, years	37.5±7.5	61.3±9.0***	35.3±6.6	58.8±8.8***	35.8±8.1	58.8±8.1***
Current smokers, *n*	2	1	0	0	0	2
Body mass index, kg/m2	24.72±2.61	26.9±2.7	24.60±3.09	25.0±3.2	23.73±2.17	24.2±3.0
Systolic blood pressure, mmHg	119.8±8.9	132.2±14.7**	114.76±8.44	124.0±11.1**	118.93±12.59	125.4±14.9**
Diastolic blood pressure, mmHg	79.6±6.6	82.2±6.2	75.24±7.82	79.7±6.1	77.14±8.93	76.8±7.3
Total cholesterol, mmol/L	5.21±1.02	5.65±1.08*	4.83±0.45	5.48±0.85*	4.90±0.79	5.48±0.87*
LDL-C, mmol/L	3.30±1.04	3.61±1.06*	2.91±0.50	3.37±0.71	3.00±0.28	3.28±0.69^a^
Triglycerides, mmol/L	1.27±0.58	1.32±0.49	1.16±0.35	1.16±0.57	1.29±0.55	1.02±0.41^a^
HDL-C mmol/L	1.27±0.63	1.41±0.39	1.36±0.27	1.36±0.40	1.37±0.40	1.81±0.45*^a^
Glucose, mmol/L	4.07±1.07	5.25±1.08**	4.14±0.26	4.84±0.48*	4.11±0.61	4.73±0.33*^a^
Energy expenditure, kcal/week	1077.3±619.3	1029.3±225.3^b^	2919.3±651.3	2703.81±449.2^c^	7383.33±3761.44	4800.88±949.2**
PWC/kg, (W/kg)	2.14±0.5	1.58±0.4**	2.55±0.79	1.99±0.4*	3.18±0.4	2.59±0.5*

Data presented as mean ± SD unless otherwise stated; *p<0.05; **p<0.01; ***p<0.001 (baseline vs follow-up within the same LTPA category); ^a^ *p*<0.05 (low-to-moderate vs very high LTPA group at follow-up); ^b^ *p*<0.001 (low-to-moderate vs very high LTPA group at follow-up) ^c^ *p*<0.01 (high vs very high LTPA group at follow-up)

Abbreviations: *LDL-C* low density lipoproteins, *HDL-C* high density lipoproteins, *PWC* physical working capacity

While most of the parameters worsened, high-density lipoproteins concentration increased and TG decreased during the observation, especially among the most active men (*p* < 0.05). Diastolic blood pressure and low-density lipoproteins remained fairly stable between baseline and follow-up examination. At follow-up the group with very high LTPA level had significantly lower low-density lipoproteins, triglycerides, glucose, and higher high-density lipoproteins than the cohort with the low-to moderate LTPA group (Table 2). There were no statistically significant differences in terms of prevalence of smoking and alcohol consumption between the studied cohorts at follow-up.

Table 3 shows the relationships between novel biochemical markers and indicators of subclinical atherosclerosis in the whole studied group (*n* = 62). Positive correlation was observed between hsCRP and IMT (*p* < 0.01), and irisin and IMT (*p* < 0.05). No significant correlations were found between CAC and either of the analyzed biomarkers. Aerobic capacity was inversely related to plasma irisin (*p* < 0.05).

**Table 3:** Relationships between biomarkers and indices of subclinical atherosclerosis among men with stable physical activity level during 25-year of observation

	Coronary artery calcification (Agatston units)	Carotid intima-media thickness (mm)	Physical working capacity (W/kg)
hsCRP, mg/L	0.039	0.301**	-0.186
Homocysteine, μmol/lL	-0.052	-0.096	0.202
Oxidized-LDL, ng/mL	-0.069	-0.006	0.149
ICAM-1, ng/mL	0.180	0.012	0.021
VCAM-1, ng/mL	-0.026	-0.173	0.098
Interleukine-6, pg/mL	0.087	0.040	0.065
Leptin, ng/mL	-0.070	-0.122	-0.064
Resistin, ng/mL	-0.017	-0.175	0.147
Adiponectin, μmol/L	-0.091	-0.053	0.192
Irisin, μmol/L	-0.032	0.223*	-0.261*

* *p* < 0.05 ** *p* < 0.01; Abbreviations: *hsCRP* high sensitivity C-reactive protein, *ICAM-1* soluble intracellular adhesion molecule-1, *sVCAM-1* soluble vascular cell adhesion molecule-1

Regarding endothelial function, a significant negative relationship was noted between RHI and Il-6 (*p* < 0.01), s-ICAM and leptin (*p* < 0.05) (data not shown in the tables).

Distribution of the mean values of biomarkers’ levels according to the long-term LTPA patterns is showed in Table 4. When comparing the cohorts with maintained LTPA level, the lowest hs-CRP, ox-LDL, leptin and irisin concentrations were found among men with stable high LTPA (2050-3840 kcal/week). Borderline correlation (*p* = 0.0503) occurred in the analysis of Interleukine-6 across the three groups. The highest prevalence of negative CAC, no cases of increased IMT and decreased RHI were observed in the group with maintained high LTPA (2050-3840 kcal/week) (Table 4).

**Table 4:** Distribution of novel biochemical markers and indices of atherosclerosis among men with stable physical activity level patterns during 25 year-observation

	Stable physical activity level during the 25-year observation		
	Low-to-moderate (<2050 kcal/week)	High (2050-3840 kcal/week)	Very high (>3840 kcal/week)
	*n*=26	*n*=21	*n*=15
Age, years	61.3 ± 9.0	58.8 ± 8.8	58.8± 8.1
hsCRP, mg/L	2.93 ± 1.1	2.20 ± 1.0*	2.82 ± 1.3
Homocysteine, μmol/L	13.55 ± 9.0	14.75 ± 4.8	14.6 ± 5.5
Oxidized-LDL, ng/mL	119.76 ± 252.3	68.35 ± 67.7*	119.48 ± 113.4
(median)	(56.80)	(13.9)	(49.50)
ICAM-1, ng/mL	498.77 ± 139.2	460.00 ± 148.8	511.2 ± 102.1
VCAM-1, ng/mL	698.55 ± 328.3	743.05 ± 272.4	617.63 ± 260.3
Interleukine-6, pg/mL	2.58 ± 5.6	1.02 ± 0.9^a^	2.88 ± 2.8
Leptin, ng/mL	9.10 ± 8.1	4.71 ± 3.07*	7.79 ± 5.18
Resistin, ng/mL	4.57 ± 1.8	4.99 ± 2.48	4.50 ± 1.42
Adiponectin, μmol/L	8.13 ± 2.8	8.99 ± 2.6	7.73 ± 4.4
Irisin, μmol/L	0.54 ± 0.14	0.47 ± 0.13*	0.48 ± 0.22
Coronary artery calcium	286.1 ± 361.9	10.7 ± 28.9	106.1 ± 278.3
(median)	(121.3)	(1.7)***	(6.30)
0, *n*	1	10**	6
Intima-media thickeness, mm	0.751 ± 0.19	0.641 ± 0.26^b^	0.750 ± 0.60
>0.9, *n*	5	0	1
Reactive hyperemia index	1.69 ± 0.4	2.00 ± 0.4	2.13 ± 0.5
<1.67, *n*	12	0**	3

Data presented as mean ± SD unless otherwise stated; *p<0.05; ** *p*<0.01; ***p<0.001; ^a^ *p*=0.0503; ^b^ *p*=0.0502. Abbreviations as in Table 2

Additional analysis was performed among men who completed at least one marathon run during preceding 5 years (*n* = 5). As compared with other highly active individuals, the most striking findings comprised the prevalence of advanced calcification (four cases of CAC ≥ 100 AU) and higher plasma concentration of hs-CRP (3.12 ± 1.4 mg/L), oxidized-LDL (249.8 ± 129 ng/ml), Interleukine-6 (3.74 ± 2.4 pg/ml) (data not shown in the tables).

## Discussion

The current study provided a comprehensive assessment of cardiovascular biomarkers in relation to lifetime stable physical activity patterns in asymptomatic middle-aged men. To our knowledge this is the first research investigating the relationship between a wide range of novel biomarkers and recognized measures of atherosclerosis in the unique cohort of men with stable LTPA level tracked prospectively for a period of about 25 years.

Of note, the mean exercise-related energy expenditure fell in all three groups over time, the statistically significant reduction in the highest LTPA level group. Probably, the age-related physiological decline in the possibilities for performing exhaustive exercises resulted in substantial reduction of EE in this cohort at follow-up.

The obtained results revealed that the most beneficial profile of biomarkers were observed in the cohort representing stable high LTPA level (i.e. energy expenditure of 2050-3840 kcal/week). Higher LTPA level was associated with less favorable biochemical status and did not differ substantially from the results obtained in the low-to-moderate LTPA group.

Interestingly, low-to moderate LTPA level occured not be sufficient to maintain fairly stable profile of traditional risk factors. This group of men developed more cardiometabolic disorders which could contribute to worse profile of novel biomarkers and indices of atherosclerosis. However, the most favorable CVD profile was observed among participants with the highest LTPA. Therefore we could expect that also profile of novel biomarkers and indices of atherosclerosis would be substantially better as compared to lower activity groups. It seems that regular PA level was a crucial mechanism, independent of other analyzed CVD risk factors, in modifying subclinical atherosclerosis.

It is postulated that cardioprotective effects of physical activity may result from affecting key inflammatory biomarkers. Acute bouts of exercise initially activates secretion of large doses of cytokines inducing a transient, mostly proinflammatory status. However, a regular training may result in a chronic anti-inflammatory response [11, 12]. The results of studies investigating the relationship between systematic exercise and cardiovascular biomarkers are, nevertheless, inconsistent. While most studies demonstrated inverse linear relationship between exercise and inflammatory biomarkers [2, 19–21], others did not confirm such associations [22, 23]. In our physically active cohorts, we did not find linear relationships between LTPA level and analyzed biomarkers. However, significantly lower concentrations of hsCRP, oxidized-LDL, Interleukine-6 and leptin were found in the cohort with the intermediate LTPA level (energy expenditure of 2050-3840 kcal/week).

This cohort also had significantly fewer cases of positive CAC, increased IMT and endothelial dysfunction. Therefore, it is possible that such a level of LTPA may protect against atherosclerosis through beneficial modification of biomarkers involved in inflammation and lipids oxidation.

Some prior studies indicated that excessive exercises might induce deleterious effects on several inflammatory markers, atherosclerosis indices and overall morbidity [24–28]. It has been demonstrated that high-intensity prolonged exercise may cause an acute pro-inflammatory response and substantial increase in inflammatory markers or endothelial progenitors [24–26]. There is an increasing body of evidence that the atherogenic effect of excessive exercise could be explained, at least in part, by increased oxidative stress, low-grade systemic inflammation and endothelial dysfunction. The results obtained in our study confirm, to some extent, the above findings indicating increased low-grade inflammation in subjects with lifetime very high LTPA level. Moreover, markedly increased concentrations of hs-CRP, oxidized-LDL, Interleukine-6 and positive CAC among regular marathon runners are consistent with some previous reports demonstrating that habitual high-intensity exercise does not reduce the magnitude of subclinical atherosclerosis [29, 30]. These findings raise concerns and require further investigations in order to distinguish not only sufficient, but also a safe threshold of physical activity level.

In the available literature there are inconsistencies concerning the association between PA and biomarkers of oxidative stress and inflammation. Some authors [31] showed an inverse relationship between PA and blood ox-LDL levels, while others failed to document any correlations or significant results were limited to selected populations [32, 33]. In our study we have not found an inverse relationship between lifetime LTPA level and oxidative stress. Regular low-to-moderate PA as well as excessive (very high) PA occurred less beneficial than high PA level. Similar patterns were observed for hs-CRP, Il-6 and indices of subclinical atherosclerosis. It seems that energy expenditure below 2000 kcal may not be sufficient in prevention of atherosclerosis. The probable explanation of these results might be the fact that the cohort with the lowest LTPA level developed substantially more metabolic disorders than the high LTPA group. It has been shown that metabolic disorders are associated with excess of reactive oxygen species and pro-inflammatory status [34]. Based on our results, exercise-related EE below 2050 kcal/week is not enough to maintain metabolically healthy profile through middle adulthood. Consequently, this level of physical activity may not prevent age-dependent deterioration in inflammatory and oxidative stress system It seems that many fundamental details of mechanisms linking dose-response exercise to altered oxidative stress and inflammation have yet to be discovered.

It might have been expected that advanced coronary calcium, carotid IMT and endothelial dysfunction would be related to less favorable status of analyzed biomarkers. Indeed, we found significant correlations between IMT, hs-CRP and irisin as well as between RHI, Il-6, s-ICAM and leptin. However, no evident relationships between CAC and either of the analyzed biomarkers were noted.

There are several studies demonstrating the association between inflammation and atherosclerosis [8–10]. The CRP level has been shown to independently predict future cardiovascular events and seems to have the most established position in prevention and management of cardiovascular diseases. However, studies analyzing the association between hs-CRP and subclinical atherosclerosis produced conflicting results [34–38]. In the present study we observed a strong positive association between hs-CRP and IMT which is in line with other studies indicating the important role of hs-CRP in the development of carotid atherosclerosis [35, 36].

Regarding the relationship between CAC and inflammatory markers, correlations found in several previous studies were no longer significant after adjusting for adiposity [5, 36, 38–40]. In our study, the lack of significant relationship between CAC and the analyzed biomarkers may be partially associated with a very low proportion of men with excessive weight. Blaha et al. (2011) showed that obesity was strongly associated with CAC and IMT independently of hsCRP, while in the absence of obesity, hsCRP was not associated with IMT [36]. It seems that CAC and hs-CRP are closely related with adiposity and might provide independent information on cardiovascular risk.

Little is known about the relationship between LTPA, subclinical atherosclerosis and biomarkers involved in regulating energy balance, like leptin or irisin. Irisin, a novel myokine, is thought to be able to regulate cardio-metabolic parameters and potentially influence atherosclerogenesis [41]. Training-induced changes in serum concentrations of irisin remain still undefined. First reports showed that endurance exercise in healthy adults may increase plasma irisin as compared to the baseline state [42, 39]. A few further studies could not confirm any relationship between circulating irisin and physical activity level [43]. In our analysis, we found a strong inverse relationship between serum irisin and aerobic capacity. These findings are consistent with the latest report of Kerstholt et al. (2015) who observed inverse associations between serum irisin and exercise capacity [44]. However, due to conflicting results of the previous studies, further investigations are needed to address this issue.

In the available literature we found only one study investigating relationship between irisin and subclinical atherosclerosis as measured by CAC or IMT. Sesti et al. (2014) reported a significant positive correlation between circulating irisin and IMT and an inverse relationship between irisin and insulin sensitivity among 192 adults [45]. The authors suggest that increased release of irisin by adipose/muscle tissue reflected a response to deterioration of insulin sensitivity or a compensatory increase to overcome an underlying irisin resistane. In our cohort, we also found a significant positive relationship between irisin and carotid IMT, but the possible explanation of this finding is difficult as the probability of insulin resistance among normal weight physically active men is very low.

Regarding endothelial function, the results obtained in the present study are consistent with the findings presented in our latest paper [13]. Both analyses demonstrated the relationship between some inflammatory markers and reactive hyperemia index. Consistently with other reports, we confirm that sICAM-1 and Interleukine-6 reflect impaired microvascular function, the initial perturbation in development of atherosclerosis [46–48].

A number of limitations of the present study should be acknowledged. As data on LTPA was self-reported, subjects might not have been accurate in estimating their actual physical activity level. However, apart from a questionnaire, an exercise test was also performed during all follow-up visits. Physical working capacity assessed during the test is thought to be a useful measure of aerobic fitness which reflects the PA level.

Due to the cross-sectional assessment of novel biomarkers and indices of atherosclerosis we could not fully determine the direction of causality. Relatively small sample size might limit statistical power for some analyses. Moreover, carotid IMT was not measured with an automatic system which might result in some variability. However, the measurements were performed with a validated tool and by an experienced operator. Among various methods to measure endothelial function, we chose the system assessing microvascular endothelial function. The endoPAT system, although not the state-of-art method, is a relatively simple, non-invasive, digital tool of assessing microvascular function by measuring postocclusive reactive hyperemia. Another recognized non-invasive method of measuring endothelial function is brachial artery flow-mediated dilation. This method is, however, technically challenging, relatively complex, and the results may be difficult to compare between clinical laboratories. Growing evidence show that both methods provide distinct information and therefore may be complementary in assessing endothelial function. Similar methodology to assess atherosclerosis has been already used in other studies [49, 50].

Despite the above limitations the study has important advantages. Long-term observation of lifestyle and traditional CVD risk, precise selection of the participants and a comprehensive assessment of a range of clinical parameters are among the most notable strengths of this study. Comparable initial cardiovascular risk, age, ethnicity, socioeconomic status, work-related PA of the studied cohorts are of particular importance.

## Conclusions

In summary, the results obtained in this analysis demonstrate that regular intense exercises substantially exceeding current recommendations may have an adverse impact on cardiovascular system. It seems that lifetime very high LTPA may exert an atherogenic effect by enhanced low-grade inflammation and lipids oxidation. There is a need to perform further prospective studies in larger samples of highly active individuals in order to establish the sufficient and safe threshold of physical activity in terms of atherosclerosis prevention.

## Availability of data and materials

Raw data supporting the obtained results are available at the corresponding author.

## Abbreviations

CAC: coronary artery calcium
CVD: cardiovascular disease
EE: energy expenditure
hsCRP: high-sensitivity C-reactive protein
Il-6: interleukine-6
IMT: intima-media thickness
LTPA: leisure-time physical activity
oxidized-LDL: oxidized low-density lipoproteins
PA: physical activity
RHI: reactive hyperemia index
sICAM-1: soluble intracellular adhesion molecule-1
sVCAM-1: soluble vascular cell adhesion molecule-1

## References

[1] Drygas W, Kostka T, Jegier A, Kuński H (2000). Long-term effects of different physical activity levels on coronary heart disease risk factors in middle-aged men. Int J Sports Med.

[2] Hamer M, Stamatakis E (2009). Physical activity and risk of cardiovascular disease events: inflammatory and metabolic mechanisms. Med Sci Sports Exerc.

[3] Rangul V, Bauman A, Holmen TL, Midthjell K (2012). Is physical activity maintenance from adolescence to young adulthood associated with reduced CVD risk factors, improved mental health and satisfaction with life: the HUNT Study, Norway. Int J Behav Nutr Phys Act.

[4] Alzamora MT, Forés R, Baena-Díez JM, Pera G, Toran P, Sorribes M, PERART/ARTPER study group (2010). The peripheral arterial disease study (PERART/ARTPER): prevalence and risk factors in the general population. BMC Public Health.

[5] Hardcastle SJ, Taylor AH, Bailey MP, Harley RA, Hagger MS (2013). Effectiveness of a motivational interviewing intervention on weight loss, physical activity and cardiovascular disease risk factors: a randomised controlled trial with a 12-month post-intervention follow-up. Int J Behav Nutr Phys Act.

[6] Haskell WL, Lee IM, Pate RR, Powell KE, Blair SN, Franklin BA (2007). Physical activity and public health: updated recommendation for adults from the American College of Sports Medicine and the American Heart Association. Circulation.

[7] Kwaśniewska M, Jegier A, Kostka T, Dziankowska-Zaborszczyk E, Rębowska E, Kozińska J, Drygas W (2014). Long-term effect of different physical activity levels on subclinical atherosclerosis in middle-aged men: a 25-year prospective study. PLoS One.

[8] Arad Y, Goodman JK, Roth M, Newstein D, Guerci AD (2005). Coronary calcification, coronary disease risk factors, C-reactive protein, and atherosclerotic cardiovascular disease. J Am Coll Cardiol.

[9] Kaptoge S, Angelantonio E, Lowe G, Emerging Risk Factors Collaboration (2010). C-reactive protein concentration and risk of coronary heart disease, stroke, and mortality: an individual participant meta-analysis. Lancet.

[10] Ridker PM, Rifai N, Stampfer MJ, Hennekens CH (2000). Plasma concentration of interleukin-6 and the risk of future myocardial infarction among apparently healthy men. Circulation.

[11] Chang CL, Lee PT, Chang WT, Chang CS, Chen JH, Tsai LM (2013). The interplay between inflammation, physical activity and metabolic syndrome in a remote male geriatric community in Southern Taiwan: the Tianliao Old People (TOP) study 03. Diabetol Metab Syndr.

[12] Mora S, Cook N, Buring JE, Ridker PM, Lee IM (2007). Physical activity and reduced risk of cardiovascular events: potential mediating mechanisms. Circulation.

[13] Kwaśniewska M, Kozińska J, Dzainkowska-Zaborszczyk E, Kostka T, Jegier A, Rębowska E (2015). The impact of long-term changes in metabolic status on cardiovascular biomarkers and microvascular endothelial function in middle-aged men: a 25-year prospective study. Diabetol Metab Syndr.

[14] Fox SM, Naughton JP, Gorman PA (1972). Physical activity and cardiovascular health. II. The exercise prescription: intensity and duration. Mod Concepts Cardiovasc Dis.

[15] Gore C, Booth ML, Bauman A, Owen N (1999). Utility of pwc75% as an estimate of aerobic power in epidemiological and population-based studies. Med Sci Sports Exerc.

[16] Agatston AS, Janowitz WR, Hildner FJ, Zusmer NR, Viamonte M, Detrano R (1990). Quantification of coronary artery calcium using ultrafast computed tomography. J Am Coll Cardiol.

[17] de Groot E, Hovingh GK, Wiegman A, Duriez P, Smit AJ, Fruchart JC, Kastelein JJ (2004). Measurement of arterial wall thickness as a surrogate marker for atherosclerosis. Circulation.

[18] Rubinshtein R, Kuvin JT, Soffler M, Lennon RJ, Lavi S, Nelson RE (2010). Assessment of endothelial function by non-invasive peripheral tonometry predicts late cardiovascular adverse events. Eur Heart J.

[19] Autenrieth C, Schneider A, Döring A, Meisinger C, Herder C, Koenig W, Huber G, Thorand B (2009). Association between different domains of physical activity and markers of inflammation. Med Sci Sports Exerc.

[20] Borodulin K, Laatikainen T, Salomaa V, Jousilahti P (2006). Associations of leisure time physical activity, self-rated physical fitness, and estimated aerobic fitness with serum C-reactive protein among 3,803 adults. Atherosclerosis.

[21] Bouassida A, Chamari K, Zaouali M, Feki Y, Zbidi A, Tabka Z (2010). Review on leptin and adiponectin responses and adaptations to acute and chronic exercise. Br J Sports Med.

[22] Gray SR, Baker G, Wright A, Fitzsimons CF, Mutrie N, Nimmo MA, Collaboration SPAR (2008). The effect of a 12 week walking intervention on markers of insulin resistance and systemic inflammation. Prev Med.

[23] Jilma B, Eichler HG, Stohlawetz P, Dirnberger E, Kapiotis S, Wagner OF (1997). Effects of exercise on circulating vascular adhesion molecules in healthy men. Immunobiology.

[24] Andersson J, Jansson JH, Hellsten G, Nilsson TK, Hallmans G, Boman K (2009). Effects of heavy endurance physical exercise on inflammatory markers in non-athletes. Atherosclerosis.

[25] Goussetis E, Spiropoulos A, Tsironi M, Skenderi K, Margeli A, Graphakos S (2009). Spartathlon, a 246 km foot race: effects of acute inflammation induced by prolonged exercise on circulating progenitor reparative cells. Blood Cells Mol Dis.

[26] Kim HJ, Lee YH, Kim CK (2009). Changes in serum cartilage oligomeric matrix protein (COMP), plasma CPK and plasma hs-CRP in relation to running distance in a marathon (42.195 km) and an ultra-marathon (200 km) race. Eur J Appl Physiol.

[27] Merghani A, Malhotra A, Sharma S. The U-shaped relationship between exercise and cardiac morbidity. Trends Cardiovasc Med. 2015. doi: 10.1016/j.tcm.2015.06.005 10.1016/j.tcm.2015.06.005 26187713

[28] Wang JS, Lee T, Chow SE (1985). Role of exercise intensities in oxidized low-density lipoprotein-mediated redox status of monocyte in men. J Appl Physiol.

[29] Kröger K, Lehmann N, Rappaport L, Perrey M, Sorokin A, Budde T (2011). Carotid and peripheral atherosclerosis in male marathon runners. Med Sci Sports Exerc.

[30] Taylor BA, Zaleski AL, Capizzi JA, Ballard KD, Troyanos C, Baggish AL (2014). Influence of chronic exercise on carotid atherosclerosis in marathon runners. BMJ Open.

[31] Mansikkaniemi K, Juonala M, Taimela S, Hirvensalo M, Telama R, Huupponen R (2012). Cross-sectional associations between physical activity and selected coronary heart disease risk factors in young adults. The Cardiovascular Risk in Young Finns Study. Ann Med.

[32] Skoumas J, Pitsavos C, Panagiotakos DB, Chrysohoou C, Zeimbekis A, Papaioannou I (2003). Physical activity, high density lipoprotein cholesterol and other lipids levels, in men and women from the ATTICA study. Lipids Health Dis.

[33] Garelnabi M, Veledar E, Abramson J, White-Welkley J, Santanam N, Weintraub W, Parthasarathy S (2010). Physical inactivity and cardiovascular risk: baseline observations from men and premenopausal women. J Clin Lab Anal.

[34] Bonomini F, Rodella LF, Rezzani R (2015). Metabolic syndrome, aging and involvement of oxidative stress. Aging Dis.

[35] Baldassarre D, De Jong A, Amato M, Werba JP, Castelnuovo S, Frigerio B (2008). Carotid intima-media thickness and markers of inflammation, endothelial damage and hemostasis. Ann Med.

[36] Blaha MJ, Rivera JJ, Budoff MJ, Blankstein R, Agatston A, O’Leary DH (2011). Association between obesity, high-sensitivity C-reactive protein > =2 mg/L, and subclinical atherosclerosis: implications of JUPITER from the Multi-Ethnic Study of Atherosclerosis. Arterioscler Thromb Vasc Biol.

[37] Chapman CM, Beilby JP, McQuillan BM, Thompson PL, Hung J (2004). Monocyte count, but not C-reactive protein or interleukin-6, is an independent risk marker for subclinical carotid atherosclerosis. Stroke.

[38] Reilly MP, Wolfe ML, Localio AR, Rader DJ (2003). C-reactive protein and coronary artery calcification: The Study of Inherited Risk of Coronary Atherosclerosis (SIRCA). Arterioscler Thromb Vasc Biol.

[39] Gauss S, Klinghammer L, Steinhoff A, Raaz-Schrauder D, Marwan M, Achenbach S, Garlichs CD (2015). Association of systemic inflammation with epicardial fat and coronary artery calcification. Inflamm Res.

[40] Khera A, de Lemos JA, Peshock RM, Lo HS, Stanek HG, Murphy SA (2006). Relationship between C-reactive protein and subclinical atherosclerosis: the Dallas Heart Study. Circulation.

[41] Boström P, Wu J, Jedrychowski MP, Korde A, Ye L, Lo JC (2012). A PGC1-α-dependent myokine that drives brown-fat-like development of white fat and thermogenesis. Nature.

[42] Huh JY, Panagiotou G, Mougios V, Brinkoetter M, Vamvini MT, Schneider BE, Mantzoros CS (2012). FNDC5 and irisin in humans: I. Predictors of circulating concentrations in serum and plasma and II. mRNA expression and circulating concentrations in response to weight loss and exercise. Metabolism.

[43] Hecksteden A, Wegmann M, Steffen A, Kraushaar J, Morsch A, Ruppenthal S (2013). Irisin and exercise training in humans - results from a randomized controlled training trial. BMC Med.

[44] Kerstholt N, Ewert R, Nauck M, Spielhagen T, Bollmann T, Stubbe B (2015). Association of circulating irisin and cardiopulmonary exercise capacity in healthy volunteers: results of the Study of Health in Pomerania. BMC Pulm Med.

[45] Sesti G, Andreozzi F, Fiorentino TV, Mannino GC, Sciacqua A, Marini MA, Perticone F (2014). High circulating irisin levels are associated with insulin resistance and vascular atherosclerosis in a cohort of nondiabetic adult subjects. Acta Diabetol.

[46] Esteve E, Castro A, López-Bermejo A, Vendrell J, Ricart W, Fernández-Real JM (2007). Serum interleukin-6 correlates with endothelial dysfunction in healthy men independently of insulin sensitivity. Diabetes Care.

[47] Vita JA, Keaney JF, Larson MG, Keyes MJ, Massaro JM, Lipinska I (2004). Brachial artery vasodilator function and systemic inflammation in the Framingham Offspring Study. Circulation.

[48] Weiner SD, Ahmed HN, Jin Z, Cushman M, Herrington DM, Nelson JC (2014). Systemic inflammation and brachial artery endothelial function in the Multi-Ethnic Study of Atherosclerosis (MESA). Heart.

[49] Byrkjeland R, Stensæth KH, Anderssen S, Njerve IU, Arnesen H, Seljeflot I, Solheim S (2016). Effects of exercise training on carotid intima-media thickness in patients with type 2 diabetes and coronary artery disease. Influence of carotid plaques. Cardiovasc Diabetol.

[50] Brolin EB, Agewall S, Brismar TB, Caidahl K, Tornvall P, Cederlund K (2015). Neither endothelial function nor carotid artery intima-media thickness predicts coronary computed tomography angiography plaque burden in clinically healthy subjects: a cross-sectional study. BMC Cardiovasc Disord.

